# The mediating role of psychological resilience in the relationship between attitudes toward sport and life satisfaction

**DOI:** 10.3389/fpsyg.2026.1904263

**Published:** 2026-09-24

**Authors:** Muhammet Talha Han, Emirhan Kan, Tiago F. Venâncio, Nelson Valente, José E. Teixeira, Pedro Afonso, Ricardo Ferraz, Pedro Forte, Luís Branquinho

**Affiliations:** 1 Faculty of Sports Sciences, Karabük University, Karabük, Türkiye; 2 Faculty of Sport Sciences, Atatürk University, Erzurum, Türkiye; 3 Biosciences Higher School of Elvas, Polytechnic University of Portalegre, Portalegre, Portugal; 4 Life Quality Research Centre (LQRC-CIEQV), Santarém, Portugal; 5 SPRINT—Sport Physical Activity and Health Research and Innovation Center, Rio Maior, Portugal; 6 Training Optimization and Sports Performance Research Group (GOERD), Faculty of Sport Science, University of Extremadura, Badajoz, Spain; 7 Sport, Performance & Exercise Applied Research (SPEAR LAB), Portalegre, Portugal; 8 Department of Sport Sciences, Polytechnic University of Cávado e Ave, Guimarães, Portugal; 9 Department of Sports, Higher Institute of Educational Sciences of the Douro, Penafiel, Portugal; 10 Research Center for Active Living and Well-Being (Livewell), Polytechnic Institute of Bragança, Bragança, Portugal; 11 Department of Sport, Exercise and Health Sciences, University of Trás-os-Montes e Alto Douro, Vila Real, Portugal; 12 Research Center in Sports Sciences, Health Sciences and Human Development, Covilhã, Portugal; 13 Department of Sport Sciences, University of Beira Interior, Covilhã, Portugal; 14 Centro de Investigação do Instituto Superior de Ciência Educativas (CI-ISCE), Penafiel, Portugal; 15 Department of Sports Sciences, Polytechnic Institute of Bragança, Bragança, Portugal

**Keywords:** attitudes toward sport, life satisfaction, mediating role, psychological resilience, university students

## Abstract

**Objective:**

This study examined the association between attitudes toward sport and life satisfaction among university students and tested whether psychological resilience statistically mediated this association.

**Method:**

This cross-sectional, relational survey was conducted with 481 undergraduate students enrolled at two universities in Türkiye. Data were collected using the Attitudes Toward Sport Scale, the Life Satisfaction Scale, and the Psychological Resilience Scale–Short Form. The hypothesized indirect association was tested using Hayes' regression-based PROCESS Macro (Model 4). The statistical significance of the indirect effect was evaluated using 5,000 bootstrap resamples and 95% confidence intervals.

**Results:**

Attitudes toward sport positively and significantly predicted psychological resilience (path a: B = 0.265, SE = 0.026, β = 0.418, *p* < 0.001). Psychological resilience, in turn, positively and significantly predicted life satisfaction when attitudes toward sport was included as a predictor in the model (path b: B = 0.250, SE = 0.026, β = 0.411, *p* < 0.001). The direct association between attitudes toward sport and life satisfaction also remained statistically significant after psychological resilience was included in the model (path c′: B = 0.058, SE = 0.016, β = 0.151, *p* < 0.001). Bootstrap analysis indicated a statistically significant indirect association through psychological resilience (indirect effect = 0.066, BootSE = 0.010, 95% BootCI [0.046, 0.087]; fully standardized indirect effect = 0.172). These findings were consistent with a partial statistical mediation pattern.

**Conclusion:**

The findings suggest that more favorable attitudes toward sport are associated with higher life satisfaction both directly and indirectly through psychological resilience. However, because the study employed a cross-sectional design and did not assess actual sport participation or the basic psychological needs proposed by Self-Determination Theory, the results should be interpreted as predictive associations rather than causal effects. The study contributes to the literature by highlighting attitudes toward sport as a distinct cognitive and affective construct that may be associated with psychological resilience and life satisfaction beyond the behavioral dimension of sport participation.

## Introduction

Physical activity and sport participation have frequently been associated with physical health, psychological wellbeing, and life satisfaction (Zhang and Chen, 2019). However, sport participation and attitudes toward sport represent conceptually distinct constructs. Sport participation refers to individuals' actual behavioral engagement in sport or physical activity, whereas attitudes toward sport refer to their favorable or unfavorable cognitive and affective evaluations of sport. Within the Theory of Planned Behavior, attitude is conceptualized as an evaluative antecedent of behavioral intention and behavior, rather than as the behavior itself (Ajzen, 1991). Therefore, evidence concerning the psychological correlates of physical activity or sport participation cannot be directly generalized to attitudes toward sport.

Attitudes toward sport encompass individuals' beliefs about sport, emotional responses to sport-related experiences, and the value they attribute to sport in their lives (Koçak, 2014). An individual may hold favorable attitudes toward sport despite being unable to participate regularly because of time, health, financial, or environmental constraints. Conversely, participation may occur because of external requirements without necessarily reflecting a strongly positive attitude. This distinction indicates that attitudes may have psychological relevance independently of the frequency or intensity of actual participation. Favorable attitudes may be associated with the extent to which individuals perceive sport as meaningful, enjoyable, socially connecting, or supportive of personal development. Consistent with this possibility, Chen (2024) reported meaningful associations between athletes' attitudes toward sport and several indicators of mental health. Nevertheless, evidence specifically examining attitudes toward sport in relation to psychological resilience and life satisfaction remains limited.

The university period represents a critical developmental stage during which identity formation accelerates, life goals become clearer, and individuals encounter multiple academic, financial, and social demands (Arnett, 2000). University students frequently experience psychological distress and adjustment difficulties that may be associated with lower life satisfaction (Auerbach et al., 2018; Bewick et al., 2010; Ibrahim et al., 2013). During this period, students also develop or reconsider their orientations toward sport and active lifestyles. Accordingly, university students constitute a relevant population for examining whether the meanings and evaluations they attribute to sport are associated with psychological resources and subjective wellbeing.

Life satisfaction refers to individuals' cognitive evaluation of the overall quality of their lives according to their own criteria (Diener et al., 1985). Psychological resilience, in contrast, refers to the capacity to adapt and maintain or regain psychological functioning when confronted with stress, adversity, or changing circumstances (Connor and Davidson, 2003; Masten, 2001). Previous evidence has consistently shown that psychological resilience is positively associated with mental health and subjective wellbeing (Hu et al., 2015). Studies based on physical activity and sport participation have also reported statistical pathways involving resilience and life satisfaction (Deng et al., 2023). However, because these studies operationalized the sport-related variable as actual participation or activity level, they provide contextual rather than direct evidence for the role of attitudes toward sport.

The Theory of Planned Behavior and Self-Determination Theory provide complementary, but distinct, perspectives for interpreting the proposed relationships. The Theory of Planned Behavior explains why attitudes are relevant as cognitive and affective evaluations that may guide intentions and behavioral choices (Ajzen, 1991). However, the theory does not directly specify how attitudes may be associated with resilience or life satisfaction. Self-Determination Theory offers a complementary interpretive framework by proposing that experiences supportive of autonomy, competence, and relatedness are associated with more self-determined motivation and psychological wellbeing (Ryan and Deci, 2000). Individuals with favorable attitudes toward sport may be more likely to perceive sport as a context involving personal choice, mastery, competence, and social connection. Such perceptions may be associated with resilient self-appraisals and more positive evaluations of life.

Nevertheless, attitudes toward sport should not be treated as psychological resources that automatically produce resilience. Rather, they can be conceptualized as evaluative orientations that may be associated with how individuals interpret sport-related opportunities for mastery, agency, enjoyment, and belonging. These interpretations may coexist with psychological resilience, while resilience may, in turn, be associated with life satisfaction. Importantly, the present study did not directly measure autonomy, competence, relatedness, motivational regulation, or actual sport participation. Therefore, Self-Determination Theory is used as a theoretically relevant interpretive perspective, and the findings can only be described as consistent with its propositions rather than as direct empirical support for the theory.

Most previous studies examining sport-related factors and wellbeing have focused on behavioral indicators, such as physical activity frequency, duration, or participation status. Comparatively fewer studies have examined whether attitudes toward sport, as a distinct cognitive and affective construct, are associated with psychological resilience and life satisfaction within an integrated model. Examining the attitudinal dimension may therefore contribute to the literature by providing a perspective that complements, rather than replaces, existing behavioral research. It may also clarify whether the value and meaning attributed to sport are statistically associated with wellbeing-related outcomes even when actual participation is not the focal predictor.

Accordingly, the present study aimed to examine the predictive associations among attitudes toward sport, psychological resilience, and life satisfaction and to test whether psychological resilience statistically mediated the association between attitudes toward sport and life satisfaction. The following hypotheses were formulated:

H1: Attitudes toward sport are positively associated with psychological resilience.
H2: Psychological resilience is positively associated with life satisfaction.
H3: Attitudes toward sport are positively associated with life satisfaction.
H4: Psychological resilience statistically mediates the association between attitudes toward sport and life satisfaction.

## Method

### Research model

Procedurally, this study was designed as a cross-sectional, relational survey to capture a snapshot of the current predictive pathways without claiming causality (Christensen et al., 2011). Within this operational framework, the direct and indirect relationships among Attitudes toward Sport, Psychological Resilience, and Life Satisfaction were systematically modeled. Finally, for the statistical evaluation phase, the regression-based PROCESS Macro (Model 4) software developed by Hayes (2022) was employed to compute and test these specific mediational effects. The conceptual model is presented in Figure 1.

**Figure 1:**
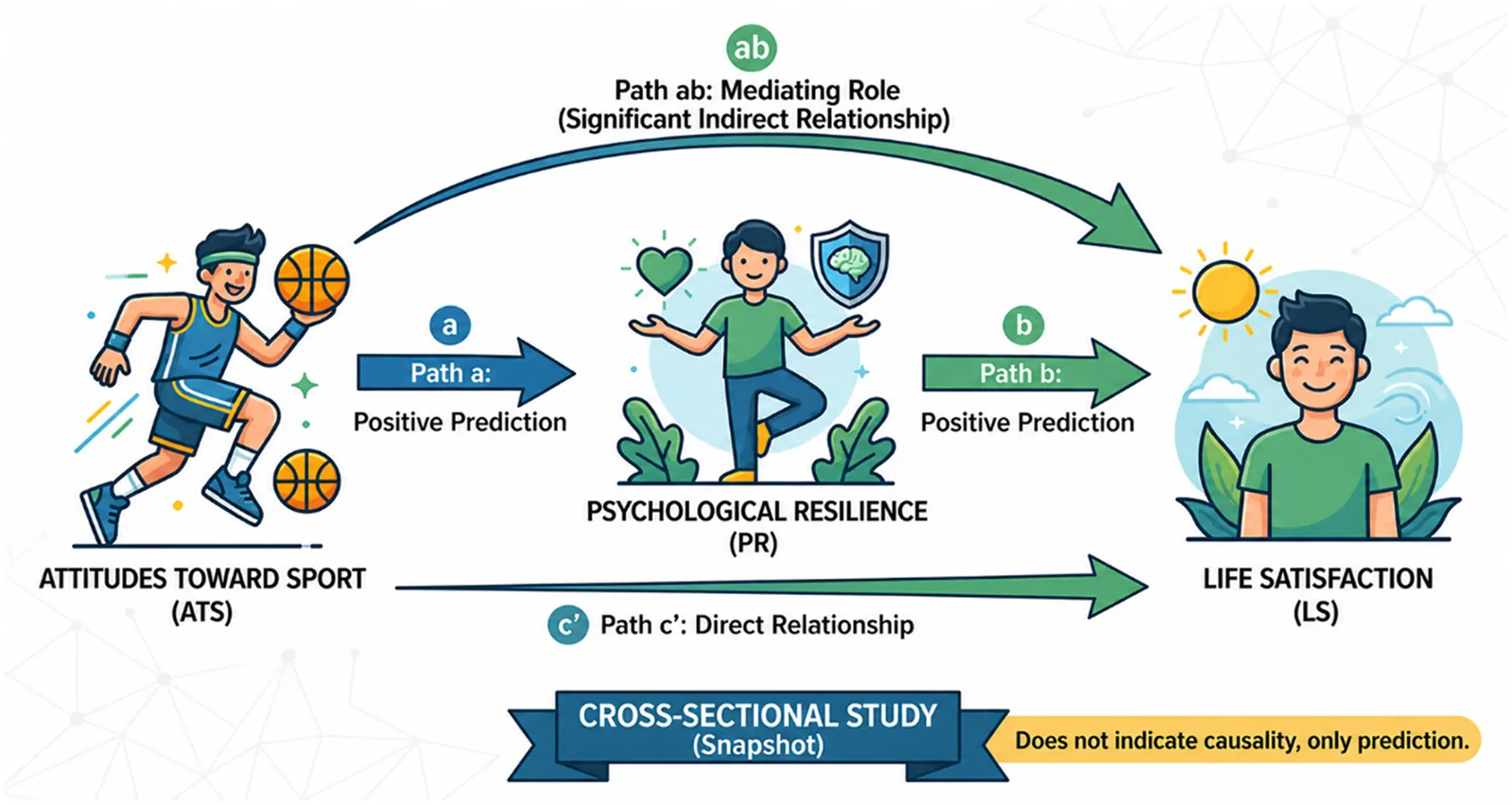
Conceptual model of the mediating role of psychological resilience in the relationship between attitudes toward sport and life satisfaction. This figure presents the statistical pathways tested in the mediation model. Attitudes toward Sport were positively associated with Psychological Resilience through path a, while Psychological Resilience was positively associated with Life Satisfaction through path b. The indirect pathway (ab) indicated a statistically significant indirect association between Attitudes toward Sport and Life Satisfaction through Psychological Resilience. The direct pathway between Attitudes toward Sport and Life Satisfaction (c′) also remained statistically significant after Psychological Resilience was included in the model, indicating a partial statistical mediation pattern. Because the study employed a cross-sectional design, the directional arrows represent theoretically specified predictive associations and do not establish temporal ordering or causal effects.

### Study group

The accessible population of the study consisted of undergraduate students enrolled at Atatürk University and Karabük University. Given the study's theoretical framework, which identifies the university years as a critical developmental stage characterized by multidimensional stressors, this demographic was purposively selected as an optimal context to examine the mechanisms of psychological resilience. To ensure the methodological rigor and homogeneity of the sample, strict inclusion and exclusion criteria were established.

*Inclusion Criteria:* (1) Being actively enrolled in an undergraduate program at a university in Türkiye, (2) being between the ages of 18 and 30, and (3) providing explicit voluntary consent to participate.

*Exclusion Criteria:* (1) Submitting incomplete or carelessly filled scale forms (e.g., uniform response patterns), (2) providing data that exhibit multivariate outliers during the preliminary screening phase, and (3) being enrolled in associate or graduate degree programs.

Data were initially collected from 580 participants. During data screening, *n* = 21 cases were excluded because of incomplete responses, *n* = 37 cases were excluded because of careless or invariant response patterns, and *n* = 41 cases were excluded because they were identified as multivariate outliers. No missing-value imputation was performed, and cases failing to meet the predefined criteria were removed from the dataset. Following the exclusion of 99 cases in total, the final dataset consisted of 481 participants. The sample comprised 279 women (58.0%) and 202 men (42.0%), with a mean age of 21.29 years (SD = 2.15).

To confirm the statistical adequacy of the sample size for testing the predictive pathways within the mediation model, an a priori power analysis was conducted using G^*^Power version 3.1 (Faul et al., 2009). Based on a linear multiple regression framework, an anticipated effect size of *f*^2^ = 0.05, an alpha level of 0.05, and statistical power of 0.95, the minimum required sample size was calculated as 312. Consequently, the final sample of 481 participants exceeded the required sample size for testing the research hypotheses.

### Data collection instruments

In addition to a personal information form, three different measurement instruments were used in the study to assess the main variables:

*Attitudes Toward Sport Scale:* This scale was developed by Koçak (2014) to measure individuals' multidimensional attitudes toward sport within the Turkish cultural context. The measurement model consists of 22 items evaluated on a 5-point Likert-type scale ranging from 1 (strongly disagree) to 5 (strongly agree). A representative item from the scale is “Participating in sports increases success in social life.” Previous studies have demonstrated that the scale is a valid and reliable measurement tool for Turkish samples.

*Life Satisfaction Scale:* This scale was developed by Diener et al. (1985) and adapted into Turkish by Dagli and Baysal (2016) to determine individuals' levels of life satisfaction. The measurement model consists of 5 items evaluated on a 5-point Likert-type scale ranging from 1 (strongly disagree) to 5 (strongly agree). A representative item from the scale is “If I could live my life over, I would change almost nothing.” Previous studies have demonstrated that the scale is a valid and reliable measurement tool for Turkish samples.

*Psychological Resilience Scale—Short Form:* This scale was originally developed by Connor and Davidson (2003), shortened by Campbell-Sills and Stein (2007), and adapted into Turkish by Koçak (2014) to assess individuals' psychological resilience. The measurement model consists of 10 items evaluated on a 5-point Likert-type scale ranging from 1 (strongly disagree) to 5 (strongly agree). A representative item from the scale is “I am able to adapt when changes occur.” Previous studies have demonstrated that the scale is a valid and reliable measurement tool for Turkish samples.

Before proceeding to the main hypothesis testing, a series of preliminary analyses were conducted to verify the structural validity, reliability, and statistical suitability of the data collection instruments and the dataset. In this context, Confirmatory Factor Analysis (CFA) fit indices, convergent validity (AVE), Composite Reliability (CR), and internal consistency (Cronbach's Alpha) coefficients were calculated. Additionally, the assumption of normality and the potential risk of Common Method Bias (CMB) arising from self-report measures were tested. The statistical parameters related to these analyses are presented in Table 1.

**Table 1:** Confirmatory Factor Analysis (CFA), validity, and reliability findings for the measurement models.

Indices/values	Attitudes toward sport	Psychological resilience	Life satisfaction
*X^2^/df*	3.72	3.97	4.74
GFI	0.86	0.95	0.98
CFI	0.88	0.95	0.98
RMSEA	0.07	0.08	0.08
SRMR	0.06	0.04	0.03
Cronbach's alpha	0.93	0.89	0.85
Composite Reliability (CR)	0.94	0.89	0.85
Average Variance Extracted (AVE)	0.43	0.45	0.54
Skewness	−0.332	−0.317	−0.012
Kurtosis	0.511	−0.444	−0.491

The findings presented in Table 1 provided mixed evidence regarding the measurement quality of the instruments. The RMSEA and SRMR values were within acceptable ranges for all three measurement models, and the Composite Reliability and Cronbach's Alpha coefficients indicated adequate internal consistency. However, the CFI (0.88) and GFI (0.86) values obtained for the Attitudes Toward Sport Scale were below the conventionally recommended 0.90 criterion. In addition, the Average Variance Extracted (AVE) values for the Attitudes Toward Sport Scale (0.43) and Psychological Resilience Scale (0.45) were below the recommended 0.50 level. Although the adequate reliability coefficients partially support the consistency of the scores, they do not eliminate the concerns regarding model fit and convergent validity. Therefore, the psychometric findings for these two instruments should be interpreted cautiously and are acknowledged as measurement-related limitations of the study. Finally, the skewness and kurtosis values were within the ±1.0 range, indicating that the observed scores did not show substantial deviations from univariate normality (Hair et al., 2014).

Given that the research data were collected using a cross-sectional, self-report method, the potential risk of Common Method Bias (CMB) was evaluated. To determine whether a single factor artificially inflated the relationships among variables, Harman's Single-Factor Test was executed. The unrotated principal component analysis revealed that the single largest factor accounted for only 29.36% of the total variance. Because this value is substantially below the critical 50% threshold, it provides preliminary diagnostic evidence that CMB is not a pervasive issue capable of invalidating the statistical results of the study (Podsakoff et al., 2003). Taken together, these findings suggested that common method bias was unlikely to fully account for the observed relationships. Nevertheless, this diagnostic result does not eliminate all sources of common method variance, and the measurement-related limitations concerning model fit and convergent validity should be considered when interpreting the results.

### Ethical procedure

This study was conducted in full compliance with the ethical guidelines of the Declaration of Helsinki, as well as the fundamental principles of scientific research and publication ethics. Prior to the commencement of the study, the research protocol was thoroughly reviewed and unanimously approved by the Social and Human Sciences Research Ethics Committee of Karabük University (Meeting Date: 26.12.2025; Session No: 2025/12; Decision No: 89). Prior to the data collection phase, all participants were comprehensively informed about the study's purpose, and explicit informed consent was obtained through a “Voluntary Participation Form.” The anonymity of the participants and the confidentiality of the collected data were strictly guaranteed throughout all stages of the research.

### Data collection

Data were collected between January and February 2026 from undergraduate students enrolled at Atatürk University and Karabük University. The two universities were selected purposively, whereas individual participants were recruited through convenience sampling on a voluntary basis. The scale battery was administered face-to-face in paper-and-pencil format in classroom settings. The instruments were presented in the following order: Personal Information Form, Attitudes Toward Sport Scale, Life Satisfaction Scale, and Psychological Resilience Scale–Short Form. Completion of the questionnaire battery took approximately 12–15 min.

Before administration, the participants were informed about the purpose of the study, voluntary participation, confidentiality, and their right to withdraw without providing a reason. The researchers were present during administration to provide standardized instructions and respond to procedural questions; however, they did not intervene in participants' responses. No personally identifying information was collected. Completed forms were stored securely and subsequently entered into the electronic dataset for screening and analysis.

### Data analysis

The data were entered into Microsoft Excel and subsequently transferred to IBM SPSS Statistics version 27 (IBM Corp., Armonk, NY, United States) for screening and statistical analysis. The mediation analysis was conducted using PROCESS Macro version 5.0 (developed by Andrew F. Hayes, Calgary, AB, Canada). Analyses were performed using the final dataset of 481 participants.

Before hypothesis testing, the data were examined for missing values, careless response patterns, univariate normality, multivariate outliers, and multicollinearity. Skewness and kurtosis values were used to evaluate univariate normality. Multivariate outliers were evaluated using Mahalanobis distance, with cases exceeding the critical chi-square value at *p* < 0.001 considered for exclusion. Multicollinearity was assessed using tolerance and Variance Inflation Factor (VIF) values. Tolerance values were above 0.20, and VIF values were below the recommended thresholds, indicating that multicollinearity was not a substantial concern (Hair et al., 2014; Kutner et al., 2005).

Hayes' PROCESS Model 4 was used to test the hypothesized indirect association (Hayes, 2022). Attitudes toward sport scores were specified as the predictor variable (X), psychological resilience scores as the mediator variable (M), and life satisfaction scores as the outcome variable (Y). The indirect effect was evaluated using 5,000 bootstrap resamples and a 95% confidence interval. An indirect effect was considered statistically significant when the bootstrap confidence interval did not include zero. Unstandardized regression coefficients (B) were reported for the estimated pathways, while standardized regression coefficients (β) were additionally presented to facilitate the interpretation of relative effect magnitudes.

## Findings

In this section, the results of the statistical analyses testing the research hypotheses are presented. The findings are reported in two systematic stages. First, the descriptive statistics and bivariate relationships among the study's main variables—Attitudes toward Sport, Psychological Resilience, and Life Satisfaction—are presented via correlation analysis. Second, the predictive parameters and specific pathways of the conceptual PROCESS model, which was established to test the mediating role of Psychological Resilience, are detailed.

As shown in Table 2, participants reported high levels of Attitudes toward Sport (M = 93.44, SD = 10.71) and moderately high levels of both Psychological Resilience (M = 37.98, SD = 6.80) and Life Satisfaction (M = 17.03, SD = 4.13). Furthermore, all main study variables were found to be positively and significantly correlated. Specifically, Attitudes toward Sport exhibited a moderate positive relationship with both Psychological Resilience (*r* = 0.418, *p* < 0.01) and Life Satisfaction (*r* = 0.324, *p* < 0.01). A moderate-to-strong positive association was also observed between Psychological Resilience and Life Satisfaction (*r* = 0.475, *p* < 0.01). These significant bivariate associations provide a robust empirical foundation for testing the regression-based mediational pathways (paths a, b, and c) within the conceptual model.

**Table 2:** Correlation coefficients among the research variables.

Variables	Mean	SD	1	2	3
1. Attitudes toward sport	93.44	10.71	1		
2. Psychological resilience	37.98	6.80	0.418^**^	1	
3. Life satisfaction	17.03	4.13	0.324^**^	0.475^**^	1

*N* = 481. ^**^p < 0.01.

As shown in Table 3, the sequential PROCESS analysis indicated that all specified statistical pathways in the mediation model were significant.

**Table 3:** Model parameters for the mediating role of psychological resilience in the relationship between attitudes toward sport and life satisfaction.

Regression models	B	SE	β	*t*	*p*	95% LLCI	95% ULCI
Model 1: prediction of the mediator variable							
Constant	13.164	2.479		5.309	<0.001	8.292	18.035
Attitudes toward sport → psychological resilience (path a)	0.265	0.026	0.418	10.076	<0.001	0.213	0.317
*R^2^ = 0.174, F_(1, 479)_ = 101.53, p <0.001*							
Model 2: prediction of the dependent variable (total effect)							
Constant	5.368	1.568		3.422	<0.001	2.286	8.450
Attitudes Toward Sport → Life Satisfaction (path c)	0.124	0.016	0.323	7.485	<0.001	0.092	0.157
*R^2^ = 0.104, F_(1, 479)_ = 56.03, p <0.001*							
Model 3: prediction of the dependent variable (direct effect)							
Constant	2.078	1.484		1.400	0.162	−0.838	4.994
Attitudes toward sport → life satisfaction (path c′)	0.058	0.016	0.151	3.461	<0.001	0.025	0.091
Psychological resilience → life satisfaction (path b)	0.250	0.026	0.411	9.403	<0.001	0.197	0.302
*R^2^ = 0.244, F_(2, 478)_ = 77.34, p <0.001*							
**Indirect path**	**Effect**	**BootSE**	**Fully standardized indirect effect**		**BootLLCI**		**BootULCI**
Through psychological resilience (path ab)	0.066	0.010	0.172		0.046		0.087

*N* = 481. B, unstandardized regression coefficient; SE, standard error; β, standardized regression coefficient; LLCI, lower limit of the confidence interval; ULCI, upper limit of the confidence interval; BootSE, bootstrap standard error; BootLLCI, lower limit of the bootstrap confidence interval; BootULCI, upper limit of the bootstrap confidence interval. The value reported for the indirect pathway is the unstandardized indirect effect; the fully standardized indirect effect is reported separately. The statistical pathways do not establish causal relationships.

In Model 1, Attitudes toward Sport positively and significantly predicted Psychological Resilience (path a: B = 0.265, SE = 0.026, β = 0.418, *t* = 10.076, *p* < 0.001, 95% CI [0.213, 0.317]). The model explained 17.4% of the variance in Psychological Resilience, *R*^2^ = 0.174, *F*_(1, 479)_ = 101.53, *p* < 0.001.

In Model 2, Attitudes toward Sport positively and significantly predicted Life Satisfaction, representing the total association between the predictor and outcome variables (path c: B = 0.124, SE = 0.016, β = 0.323, *t* = 7.485, *p* < 0.001, 95% CI [0.092, 0.157]). Attitudes toward Sport alone explained 10.4% of the variance in Life Satisfaction, *R*^2^ = 0.104, *F*_(1, 479)_ = 56.03, *p* < 0.001.

In Model 3, Attitudes toward Sport and Psychological Resilience were simultaneously included as predictors of Life Satisfaction. The model explained 24.4% of the variance in Life Satisfaction, *R*^2^ = 0.244, *F*_(2, 478)_ = 77.34, *p* < 0.001. Psychological Resilience positively and significantly predicted Life Satisfaction (path b: B = 0.250, SE = 0.026, β = 0.411, *t* = 9.403, *p* < 0.001, 95% CI [0.197, 0.302]). The direct association between Attitudes toward Sport and Life Satisfaction also remained statistically significant after Psychological Resilience was included in the model (path c′: B = 0.058, SE = 0.016, β = 0.151, *t* = 3.461, *p* < 0.001, 95% CI [0.025, 0.091]).

The bootstrap analysis based on 5,000 resamples indicated a statistically significant indirect association between Attitudes toward Sport and Life Satisfaction through Psychological Resilience (indirect effect = 0.066, BootSE = 0.010, 95% BootCI [0.046, 0.087]). The fully standardized indirect effect was 0.172. Because the bootstrap confidence interval did not include zero and the direct association remained statistically significant after Psychological Resilience was included in the model, the findings were consistent with a partial statistical mediation pattern.

## Discussion

The present study examined the predictive associations among attitudes toward sport, psychological resilience, and life satisfaction and tested whether psychological resilience statistically mediated the association between attitudes toward sport and life satisfaction. The findings indicated that more favorable attitudes toward sport were associated with higher psychological resilience and life satisfaction and that psychological resilience statistically accounted for part of the association between attitudes toward sport and life satisfaction. Because the data were cross-sectional, the findings represent associations observed at a single point in time and do not establish the temporal or causal direction of the relationships.

The positive association between attitudes toward sport and psychological resilience supported the first hypothesis. This finding suggests that students who evaluated sport more favorably also tended to report a greater perceived capacity to adapt to difficulties and stressful circumstances. Chen (2024) similarly reported associations between attitudes toward sport and mental health indicators among athletes. However, much of the broader literature concerns sport participation, physical activity levels, or characteristics of the sport environment rather than attitudes toward sport. For example, Zhang and Li (2025) examined sport atmosphere, whereas Deng et al. (2023) examined physical activity. These findings provide relevant contextual comparisons, but they should not be treated as direct evidence concerning the attitudinal construct examined in the present study.

The distinction between attitudes and participation is theoretically important. Within the Theory of Planned Behavior, attitudes represent favorable or unfavorable evaluations that may contribute to intentions and behavior, but they are not equivalent to actual behavioral engagement (Ajzen, 1991). The present findings therefore suggest that the cognitive and affective meanings attributed to sport may have psychological relevance in addition to the behavioral dimension of participation. However, the observed association may also reflect factors that were not measured in the current study. Students with greater previous sport experience, stronger self-efficacy, better physical health, higher social support, or more favorable socioeconomic conditions may report both more positive attitudes toward sport and greater resilience. Reverse directionality is also possible, as psychologically resilient individuals may develop more favorable evaluations of sport-related challenges and experiences.

Self-Determination Theory provides a possible, but indirect, perspective for interpreting this association. Individuals who perceive sport as a context involving personal choice, mastery, competence, and social connection may evaluate sport more positively, and these perceptions may be associated with adaptive psychological functioning (Ryan and Deci, 2000). Nevertheless, autonomy, competence, relatedness, and motivational regulation were not measured in the present study. Accordingly, the findings should not be interpreted as demonstrating that positive attitudes toward sport satisfy basic psychological needs or directly develop resilience. They can only be considered broadly consistent with the possibility that favorable sport-related evaluations coexist with psychologically adaptive orientations.

Psychological resilience was also positively associated with life satisfaction, supporting the second hypothesis. This result is consistent with previous research identifying resilience as an important correlate of mental health and subjective wellbeing (Hu et al., 2015). Individuals who perceive themselves as better able to adapt to stressful conditions may also evaluate their lives more positively. At the same time, the cross-sectional design does not demonstrate that resilience precedes life satisfaction. Higher life satisfaction may facilitate more resilient self-evaluations, or both constructs may be influenced by common factors such as social support, personality characteristics, psychological health, financial security, and the quality of interpersonal relationships.

Attitudes toward sport were also positively associated with life satisfaction, supporting the third hypothesis. However, attitudes toward sport alone explained 10.4% of the variance in life satisfaction. This finding suggests that favorable evaluations of sport constitute one relevant correlate of life satisfaction but are insufficient to account for the broader personal, relational, academic, and socioeconomic influences underlying individuals' evaluations of their lives. Moreover, because actual sport participation was not assessed, it remains unclear whether the association reflects attitudes independently or partly reflects unmeasured differences in previous and current sport experiences.

The amount of explained variance also requires cautious interpretation. The model including both attitudes toward sport and psychological resilience explained 24.4% of the variance in life satisfaction, indicating a meaningful but moderate level of explanatory power. In other words, approximately 75.6% of the variance in life satisfaction remained unexplained by the model. This finding is not unexpected because life satisfaction is a broad cognitive evaluation influenced by multiple personal, relational, academic, economic, and cultural factors (Diener et al., 1985). Consequently, attitudes toward sport and psychological resilience should be regarded as two relevant correlates of life satisfaction rather than as its primary or comprehensive determinants.

The statistically significant indirect association through psychological resilience supported the fourth hypothesis. More favorable attitudes toward sport were statistically associated with higher life satisfaction partly through their association with psychological resilience. However, the significant direct association that remained after psychological resilience was included in the model indicates partial rather than full statistical mediation. This pattern suggests that psychological resilience accounts for only one part of the relationship. Other variables, such as actual sport participation, positive affect, self-efficacy, sense of purpose, social belonging, and basic psychological need satisfaction, may also statistically account for the remaining direct association. The partial mediation pattern should therefore not be interpreted as evidence that attitudes toward sport causally increase resilience, which then causally increases life satisfaction. Longitudinal and experimental studies are required to evaluate temporal ordering and causal mechanisms.

The findings contribute to the literature by examining attitudes toward sport as a construct that is conceptually distinct from sport participation and physical activity frequency. Rather than demonstrating that favorable attitudes themselves constitute psychological resources, the results suggest that these attitudes are associated with resilience-related self-perceptions and life satisfaction. The findings also provide preliminary evidence for a statistical pathway involving psychological resilience in a Turkish university student sample. Nevertheless, interpretations based on Self-Determination Theory remain tentative because the theory's central constructs were not directly assessed. Future studies should simultaneously measure attitudes toward sport, actual participation, basic psychological need satisfaction, motivational regulation, and relevant contextual variables to determine whether attitudes explain unique variance beyond sport behavior.

## Conclusion

This cross-sectional study indicated that attitudes toward sport were positively associated with psychological resilience and life satisfaction among university students. Psychological resilience also statistically accounted for part of the association between attitudes toward sport and life satisfaction. Because the direct association remained significant after psychological resilience was included in the model, the findings were consistent with a partial statistical mediation pattern.

The study contributes to the literature by examining attitudes toward sport as a cognitive and affective construct that is conceptually distinct from actual sport participation. The findings suggest, rather than demonstrate, that the meanings and evaluations individuals attribute to sport may be associated with resilience-related self-perceptions and life satisfaction. However, the study does not establish that improving attitudes toward sport will cause an increase in psychological resilience or life satisfaction. Moreover, because actual sport participation, basic psychological needs, and motivational regulation were not assessed, the findings should not be interpreted as direct empirical support for Self-Determination Theory. Longitudinal and experimental research incorporating both attitudinal and behavioral sport variables is needed to clarify the temporal and explanatory processes underlying the observed associations.

## Recommendations

### Recommendations for researchers

The findings of the present study provide several suggestions for future research. First, due to the cross-sectional design of the study, causal relationships among variables cannot be established. To overcome this limitation, the use of longitudinal research designs is recommended. Longitudinal studies would provide the opportunity to examine the long-term effects of changes in attitudes toward sport on psychological resilience and life satisfaction over time.

Second, although the current model tested only the mediating role of psychological resilience, it is suggested that other psychological variables may also mediate the relationship between attitudes toward sport and life satisfaction. Examining variables such as self-efficacy (Karababa et al., 2025), positive affect (Liu et al., 2021), and mindfulness (Seçer et al., 2025) within different mediation or moderation models would provide a more comprehensive understanding of the mechanistic structure of this relationship. Third, it is recommended that the mediation structure be validated within a stronger measurement model framework using structural equation modeling (SEM) and that potential measurement errors be controlled.

Fourth, the fact that the study was conducted only with a sample of university students limits the generalizability of the findings. Comparative studies including different age groups, individuals from various occupational backgrounds, and different levels of sport participation (amateur, semi-professional, professional) are expected to make significant contributions to the literature. Finally, replicating this study conducted in a Turkish sample within a cross-cultural perspective would strengthen the validity evidence of the findings.

### Recommendations for practitioners and policy makers

The practical implications of the findings should be considered cautiously because the cross-sectional design does not demonstrate that modifying attitudes toward sport or psychological resilience will produce changes in life satisfaction. Nevertheless, universities may consider developing and evaluating student-support programs that address both sport-related beliefs and adaptive coping skills. Such programs may include opportunities for students to reflect on the personal meaning of sport, perceived barriers to participation, previous sport experiences, coping strategies, and sources of social support.

Rather than assuming that attitude-focused or resilience-focused programs will necessarily improve wellbeing, future university interventions should be evaluated through controlled, longitudinal, or pretest-posttest designs. Programs may integrate accessible sport opportunities with resilience-oriented components such as stress management, adaptive coping, goal setting, and social connectedness. Evaluating actual changes in sport participation, attitudes toward sport, psychological resilience, and life satisfaction would help determine whether these variables are causally related and which intervention components are most beneficial.

### Limitations

This study has several methodological and sample-related limitations. Clearly identifying these limitations is of great importance for the accurate interpretation of the findings and for providing a guiding framework for future research.

The first limitation relates to the research design. The study was conducted using a cross-sectional survey model. Although this design provides a snapshot of the relationships among variables, it does not allow for causal inferences (Christensen et al., 2011). The question of whether attitudes toward sport increase psychological resilience or life satisfaction, or whether the process operates in the opposite direction, can only be answered through longitudinal designs. Therefore, the findings of the study should be interpreted within the framework of statistical predictive relationships and should not be used as a basis for causal conclusions.

The second limitation concerns the sampling. The data were obtained from undergraduate students enrolled at two universities in Türkiye. Since participation was based on voluntariness and regional or institutional representation could not be ensured, the findings cannot be directly generalized to the entire population of Turkish university students. Studies based on probabilistic sampling methods and covering a wider geographical distribution would significantly enhance the external validity of the findings.

The third limitation stems from the use of self-report scales for data collection. Although Harman's Single-Factor Test suggested that common method bias was unlikely to fully account for the observed relationships, this diagnostic procedure cannot eliminate all possible sources of common method variance. Factors inherent in self-report measurement, such as social desirability, response tendencies, and recall-related errors, may have influenced the observed associations to some extent (Podsakoff et al., 2003). Future studies may address this limitation by combining self-report measures with behavioral observations, informant reports, or other complementary assessment methods.

The fourth limitation is related to the variables included in the research model. In examining the relationships among attitudes toward sport, psychological resilience, and life satisfaction, it should not be overlooked that other psychological variables not included in the current model may influence these relationships. For example, constructs such as personality traits, perceived social support, and self-regulation capacity may play significant moderating or mediating roles in these relationships. Studies incorporating these variables into the model are expected to contribute to a more comprehensive understanding of the underlying mechanisms (Zhang and Li, 2025).

An additional limitation concerns the distinction between attitudes toward sport and actual sport participation. Although the study focused specifically on attitudes, participants' sport participation status, frequency, duration, intensity, and previous sport experience were not assessed. Therefore, it was not possible to determine whether attitudes toward sport explained unique variance in psychological resilience and life satisfaction beyond actual behavioral engagement. It is also possible that previous or current participation influenced both attitudes toward sport and the psychological outcomes examined in the study. Future research should simultaneously include attitudinal and behavioral indicators to distinguish their independent and combined associations with wellbeing.

The theoretical interpretation based on Self-Determination Theory also requires caution. The study did not measure autonomy, competence, relatedness, intrinsic motivation, or other forms of motivational regulation. Consequently, the findings do not constitute a direct test of Self-Determination Theory and cannot demonstrate that favorable attitudes toward sport reflect basic psychological need satisfaction. The theory was used only as a complementary interpretive framework, and future studies should directly assess its central constructs.

A further limitation relates to the psychometric findings of the measurement instruments. The CFI (0.88) and GFI (0.86) values obtained for the Attitudes Toward Sport Scale were below the commonly recommended 0.90 criterion. In addition, the AVE values for Attitudes Toward Sport (0.43) and Psychological Resilience (0.45) were below the recommended 0.50 level. Although the internal consistency and Composite Reliability coefficients were adequate and the RMSEA and SRMR values were within acceptable ranges, these results indicate less-than-optimal model fit and convergent validity for two of the constructs. Accordingly, the magnitude of the observed associations should be interpreted cautiously, and future studies should further evaluate or refine the measurement models.

Finally, the fact that the Attitudes toward Sport Scale used in the study was developed specifically for the Turkish culture (Koçak, 2014) constitutes a limitation in terms of international comparative studies. To overcome issues of cultural equivalence, it is recommended that future research employ measurement instruments with established validity at the international level, which would enhance the universal validity of the findings.

## Data Availability

The raw data supporting the conclusions of this article will be made available by the authors, without undue reservation.
